# Structural intermediates of a DNA–ligase complex illuminate the role of the catalytic metal ion and mechanism of phosphodiester bond formation

**DOI:** 10.1093/nar/gkz596

**Published:** 2019-07-17

**Authors:** Adele Williamson, Hanna-Kirsti S Leiros

**Affiliations:** 1 Department of Chemistry, UiT The Arctic University of Norway, Tromsø, N-9037, Norway; 2 School of Science, University of Waikato, Hamilton 3240, New Zealand

## Abstract

DNA ligases join adjacent 5′ phosphate (5′P) and 3′ hydroxyl (3′OH) termini of double-stranded DNA via a three-step mechanism requiring a nucleotide cofactor and divalent metal ion. Although considerable structural detail is available for the first two steps, less is known about step 3 where the DNA-backbone is joined or about the cation role at this step. We have captured high-resolution structures of an adenosine triphosphate (ATP)-dependent DNA ligase from *Prochlorococcus marinus* including a Mn-bound pre-ternary ligase–DNA complex poised for phosphodiester bond formation, and a post-ternary intermediate retaining product DNA and partially occupied AMP in the active site. The pre-ternary structure unambiguously identifies the binding site of the catalytic metal ion and confirms both its role in activating the 3′OH terminus for nucleophilic attack on the 5′P group and stabilizing the pentavalent transition state. The post-ternary structure indicates that DNA distortion and most enzyme-AMP contacts remain after phosphodiester bond formation, implying loss of covalent linkage to the DNA drives release of AMP, rather than active site rearrangement. Additionally, comparisons of this cyanobacterial DNA ligase with homologs from bacteria and bacteriophage pose interesting questions about the structural origin of double-strand break joining activity and the evolution of these ATP-dependent DNA ligase enzymes.

## INTRODUCTION

DNA ligases, members of the nucleotidyl transferase superfamily, are enzymes that join juxtaposed 5′ phosphate (5′P) and 3′ hydroxyl (3′OH) termini of double-stranded DNA in a three-step catalytic mechanism which is powered by a nucleotide cofactor and requires the presence of a divalent cation. In step 1, a covalent enzyme-adenylate is formed by nucleophilic attack of a conserved lysine residue on the α-phosphate of the cofactor, which may be adenosine triphosphate (ATP) or nicotinamide adenine dinucleotide (NAD^+^), releasing either pyrophosphate or nicotinamide dinucleotide. In step 2 the adenosine monophosphate (AMP) moiety is transferred to the 5′P terminus of the DNA, activating this position for attack by the adjacent 3′OH nucleophile, which results in the new phosphodiester bond (1–3).

DNA ligases are modular enzymes comprising at a minimum, a two-domain catalytic core with the active-site bearing adenylation (AD) domain and a smaller oligonucleotide binding (OB) domain connected by a flexible linker. Progression of catalysis is accompanied by large-scale reorientation of these domains to facilitate substrate binding and product release (1). Nucleotide binding occurs in the DNA-free form and involves transient closing for formation of the AMP-lysine phosphoramidate intermediate. Subsequently the enzyme returns to an open conformation presenting an accessible binding surface for the substrate DNA, recruitment of which stimulates closing of the OB domain over the duplex forming a ternary complex with the typical C-shaped clamp required for productive phosphodiester bond catalysis (1).

The chemical transactions of ligation occur within the AD domain and are facilitated by highly conserved enzyme motifs I, III, IIIa, IV and V, that are common to all nucleotiydyl transferases (2). Crystal structures of DNA ligases in complex with cofactor and substrate have provided considerable mechanistic detail about steps 1 and 2. The adenine base of the cofactor is buried in a hydrophobic pocket sandwiched between an aliphatic residue of motif IV and aromatic sidechain from IIIa, while a conserved lysine from motif IV contacts the heterocycle N1, and in many cases an acidic residue from motif I hydrogen bonds with the extracyclic N6 (4). Throughout catalysis the position of the base remains fixed, while the ribose sugar swivels from the *syn* to *anti* configuration around the glyosidic bond. During step 1 this rotation entails a remodeling of contacts to the AMP O2′, O3′ hydroxyl groups from an acidic residue in motif III and a conserved arginine from motif I as well as interactions with a conserved lysine from motif V to O4′ (5). The greatest mobility occurs at the α-phosphate and leaving group portions of the cofactor, with the former contacted by the strictly conserved lysine of motif I, which is the site of adenylation during step 1 and a second essential lysine in motif V (4,5). In ATP-dependent ligases, residues from motif VI in the OB domain assist with reorientation of the β- and γ-phosphates, while in NAD-dependent DNA ligases this role is carried out by the dedicated 1a domain (1).

Substrate DNA binding and subsequent adenylation involves a handover of the covalent linkage of the AMP α-phosphate from the catalytic motif I lysine to the 5′P of the nick, followed by rotation of the AMP-DNA phosphoanhydride away from the catalytic lysine and toward the DNA 3′OH with a net result of returning the AMP nucleoside to a more strained *syn* configuration (6–8). Reorientation of the DNA 5′P and AMP α-phosphate are mediated by the lysine from motif V as well as the motif I catalytic lysine, while changes in non-covalent bonding to the AMP ribose hydroxyls again require rearrangement of contacts to the motif I, III and V residues. Formation of the new polynucleotide phosphodiester bond in step 3 stimulates release of both the DNA product and AMP, resetting the active site for binding of a new cofactor molecule (7).

Divalent metal cations are required for all steps of ligase catalysis. Recent structures of metal-bound RNA ligases from eukaryotes and viruses, as well as the catalytic core of the ATP-dependent DNA ligase from *Mycobacterium tuberculosis* have illuminated the location and mechanistic role of two ions in step 1 chemistry where a catalytic ion activates the lysine nucleophile and stabilizes the pentavalent transition state of the AMP α-phosphate, while a second ion serves to orient the pyrophosphate tail (9–11). NAD-dependent DNA ligases by contrast require only the catalytic ion and use the 1a domain to facilitate leaving group orientation (10).

Considerably less structural information is available for step 3 of the ligase reaction than preceding stages. Several structures of pre-step 3 enzyme–DNA complexes are known where reaction progress is blocked by use of a 3′ dideoxy terminated upstream strand (8,12,13). Progression of a DNA-bound pre-step 2 intermediate through step 3 was induced *in crystallo* by addition of Mn^2+^, but the AMP had exited the binding site of the resulting ligase-DNA product complex presumably due to complex ‘breathing’ during catalysis and no coordinated metal ions were identified (7).

In the present study we have captured high resolution structures of a DNA ligase from *Prochlorococcus marinus* (hereafter Pmar-Lig) at crucial stages of the ligase reaction including a pre-step 3 ternary complex where the catalytic metal site is occupied by manganese, and a post-ternary complex after completion of step 3 where the binding pocket is partially occupied by AMP.

Pmar-Lig is one of three ATP-dependent DNA ligases encoded in the genomes of some strains of this cyanobacterium in addition to the replicative NAD-dependent enzyme (14). ATP-dependent ligases are structurally diverse, often with additional domains appending the catalytic core of one or both termini (14,15). In some cases these modules have independent enzymatic functions (16); however the most common configuration appears to be an α-helical DNA-binding domain (DB domain) positioned N-terminal to the AD domain forming a circumferential clamp about the DNA duplex with non-covalent contacts between the DB- and OB domains completing encirclement. The recently solved structure of the T4 DNA ligase, for example, presents a compact version of this template with a 7 helix DB-domain that is structurally homologous to the central dyad of the larger mammalian and archaeal forms (8,12,13,17–19). Other DNA-encircling arrangements of ATP-dependent DNA ligases include the novel N-terminal α/β domain of the African swine fever virus (20), and the dynamic β-hairpin ‘latch’ of the *Chlorella* virus DNA ligase, which emanates from the OB domain (7). The Lig E class of bacterial ATP-dependent DNA ligases dispense with complete DNA encirclement and lack dedicated DNA-binding modules; however substrate affinity is provided by conserved motifs within the OB domain fold (6,21).

Previous bioinformatic predictions indicate that the AD and OB domains of Pmar-Lig have considerable conservation with the minimal Lig E bacterial group, while the N-terminal portion possesses moderate sequence similarity to bacteriophage ligases and was predicted to form a DNA binding domain (14). These predictions are borne out by comparison of Pmar-Lig to the subsequently published structure of T4 DNA ligase as well as recent structures of Lig E enzymes (6,12,21); thus in addition to providing insight into formation of phosphodiester bond formation during ligation, the Pmar-Lig structures contribute to our understanding of ATP-dependent DNA ligases in bacteria.

## MATERIALS AND METHODS

### Cloning and expression

Wild-type (WT) *pmar-lig*, (accession number KGF9828), as well as the point mutants *pmar-lig_R120A, pmar-lig_R120D, pmar-lig_*R120D/G359K and pmar*-lig_*C145S/C332S were ordered as codon-optimized synthetic constructs from the Thermofisher GeneArt service. Synthetic genes were supplied sub-cloned into pDONR221 and were transferred into the pDEST17 plasmid (Invitrogen) as described previously (21). Protein expression and purification of both Pmar-Lig and the ATP-dependent DNA ligase from *Alteromonas macleodii* (Ame-Lig) were carried out as previously described (21,22). T4 DNA ligase (T4-Lig) was expressed from pET14b-T4_ligase using standard conditions (induction by addition of 0.5 mM IPTG followed by incubation at 22°C overnight). Purification followed the same scheme as for Pmar-Lig and Ame-Lig, with the exception that tag removal was omitted and single round of IMAC was performed prior to gel filtration.

### Crystallization, structure determination and analysis

Single HPLC-purified oligonucleotides with the following sequences in 5′-3′ orientation were purchased from Sigma or IDT: 5′P-strand: (Phos) CAC TAT CGG AA; Complementary-strand: TTC CGA TAG TGG GGT CGC AAT; 3′OH-strand: ATT GCG A**C**C where the underlined nucleotide indicates the position where a modified 2′-O-methylcytidine was used when assembling the double-stranded substrate (2′OMe-30). In the standard substrate this position was a regular cytidine.

Oligos were resuspended at a concentration of 9 mM in annealing buffer (50 mM Tris pH 8.0, 50 mM NaCl, 1 mM ethylenediaminetetraacetic acid (EDTA)), mixed 1:1:1 to give a final duplex concentration of 3 mM and incubated at 85°C before cooling overnight.

Wild-type Pmar-Lig (370 μM) or the mutant Pmar-Lig R120A (390 μM) were incubated with 1.2 molar equivalents of nicked duplex at 4°C for ∼2 h. Pre-step 3 complexes (Pmar-Lig(PreS3), Pmar-Lig(PreS3-Mn) and Pmar-Lig(PreS3-R120A)) were incubated with the modified (2′OMe-30) substrate and included additional 5 mM EDTA during this step; the post-step 3 complex (Pmar-Lig(PostS3)) used the unmodified (standard) substrate and was not supplemented with EDTA beyond that in the incubation buffers.

Crystals were grown at 4°C by hanging drop diffusion method. Pre-step 3 forms were crystalized from 17–18% PEG 8K, 100 mM ammonium sulfate, 100 mM Bis-Tris pH 5.5. The post-step 3 was crystalized from 22% PEG 4K, 200 mM ammonium sulfate, 100 mM Bis-Tris pH 6.5.

Crystals were cryoprotected in these respective mother-liquors including 12% ethyleneglycol before flash-freezing in liquid nitrogen. To obtain the Mn^2+^-bound complex (Pmar-Lig(PreS3-Mn)), 5 mM MnCl_2_ was included in the cryoprotectant solution and the crystal was allowed to soak for ∼1 min before freezing. For Pmar-Lig(PreS3), 5mM MgCl_2_ was included in cryoprotectant and freezing was carried out immediately; however, as there was no evidence of metal ions or tightly coordinated water in this structure we concluded the soaking time was too short and have treated the resulting structure as a native complex. Longer soaking times with MgCl_2_ caused crystals to crack and diffract very poorly.

Diffraction data were measured at ESRF beamlines ID23–1 and ID30B Grenoble and BESSY, BL14.2 Berlin. Data were integrated, scaled and truncated in XDS, XSCALE (23) and AIMLESS (24). The complex structure was solved by molecular replacement using Phaser-MR (25) with the DNA–protein complex of *Alteromonas mediterranea* (PDB 6gdr) as the search model. Further refinement used the Phenix software (26) with manual rebuilding in COOT (27). CC_1/2_ values > 0.50 in the outer shell were used as the criterion for high resolution cut-off (28). Calculation of polder OMIT maps used the *phenix.polder* tool implemented in the Phenix suite (29). All structural figures were made using PyMol (Schrödinger, LLC). Data collection and statistics are listed in Table 1. Protein–DNA and inter-domain interactions were detected using the NuProPlot program (30) and the RING server (31) respectively. Metal coordination and geometry were verified with the CheckMyMetal validation tool (32).

**Table 1.** Data collection and refinement statistics output from Phenix for Pmar-Lig intermediates bound to DNA

	Native pre-step3 Pmar-Lig (PreS3)	Mn-bound pre-step3 Pmar-Lig (PreS3-Mn)	Native post-step3 Pmar-Lig (PostS3)	R120A pre-step3 Pmar-Lig (PreS3-R120A)
PDB entry	6RCE	6RAR	6RAU	6RAS
Data collection				
Resolution range (Å)	69.0–1.95 (2.05- 1.95)	68.0–1.79 (1.98–1.79)	24.17–1.99 (2.04–1.99)	24.37–2.76 (2.90–2.75)
Beamline	ESRF, ID23–1	ESRF, ID23–1	ESRF, ID30B	BESSY, BL14.2
Wavelength (Å)	0.9919	0.9919	0.9763	0.9184
Space group	P2_1_	P2_1_	P2_1_	P2_1_
Unit cell: a, b, c (Å); β (°)	67.14 103.01 68.58 91.01	67.30 103.32 68.54 90.79	66.73 102.50 68.56 91.44	67.47 103.41 68.91 90.91
Unique reflections	57 869 (2893)	57 936 (2897)	62 306 (6216)	24 531 (3557)
Multiplicity	3.4 (3.3)	3.1 (2.4)	6.6 (6.9)	5.6 (5.3)
Completeness (%)	89.0 (39.2)	88.6 (59.7)	98.7 (99.1)	99.4 (99.3)
Mean 〈I/σ_(I)_〉	10.2 (1.5)	3.1 (2.4)	9.3 (1.1)	10.0 (1.5)
Wilson B-factor (Å^2^)	32.7	29.1	36.8	43.3
R-merge	0.074 (1.946)	0.096 (0.560)	0.081 (1.154)	0.122 (0.969)
R-meas	0.088 (0.669)	0.116 (0.556)	0.099 (1.274)	0.149 (1.194)
R-pim	0.061 (0.622)	0.084 (0.425)	0.056 (0.565)	0.085 (0.687)
CC _1/2_	0.997 (0.502)	0.993 (0.604)	0.998 (0.640)	0.995 (0.572)
Resolution range in refinement (Å)	24.2–1.95 (2.02–1.95)	24.18–1.79 (1.85–1.79)	24.17–1.99 (2.06–1.99)	24.3–2.69 (2.85–2.75)
Reflections used in refinement	57 810 (1807)	57 854 (328)	62 231 (6211)	24 493 (2405)
Reflections used for R-free	2694 (58)	2876 (19)	3014 (307)	1226 (122)
R-work	0.2309 (0.3078)	0.2333 (0.3512)	0.1933 (0.2979)	0.1843 (0.2944)
R-free	0.2751 (0.3100)	0.2651 (0.3824)	0.2300 (0.3323)	0.2381 (0.3411)
Number of: non-hydrogen atoms	4891	4805	4789	4614
Protein	4373	4381	4400	4367
Ligands	26	27	15	51
Solvent	492	398	374	196
Number of protein residues	432	432	432	433
RMSD bonds (Å)	0.016	0.018	0.017	0.017
RMSD angles (°)	1.36	1.18	1.36	1.32
Ramachandran favored (%)	97.21	96.51	96.98	94.66
Ramachandran allowed (%)	2.79	3.26	3.02	5.10
Ramachandran outliers (%)	0.00	0.23	0.00	0.23
Rotamer outliers (%)	0.00	0.00	0.52	0.00
Clashscore	7.24	6.29	6.47	9.37
Average B-factor all atoms (Å^2^)	43.62	32.23	47.41	49.62
Protein (Å^2^)	43.07	31.83	47.18	49.88
Ligands (Å^2^)	34.15	27.85	68.38	55.40
Solvent (Å^2^)	49.00	37.03	49.28	42.17

### Ligation assays

Ligase activity of Pmar-Lig, Ame-Lig and T4-Lig with double- and single-stranded breaks were measured by denaturing urea-PAGE of fluorescently labeled DNA duplexes as described previously (33) (briefly, 80 nM substrate, 0.1 mM ATP, 10 mM MgCl_2_, 1.0 mM 1,4-Dithiothreitol (DTT), 100 mM NaCl, 50 mM Tris pH 8.0) and with the following assay conditions: nicked substrate 15 min at 25°C; cohesive overhang substrate 2 h 25°C; blunt substrate 18 h 15°C. Metal-dependence of ligase activity by Pmar-Lig was measured by molecular beacon assay (34) with modifications described previously (22).

## RESULTS

### The pre-ternary complex of Pmar-Lig forms an archetypal DNA clamp

**Figure 1.**
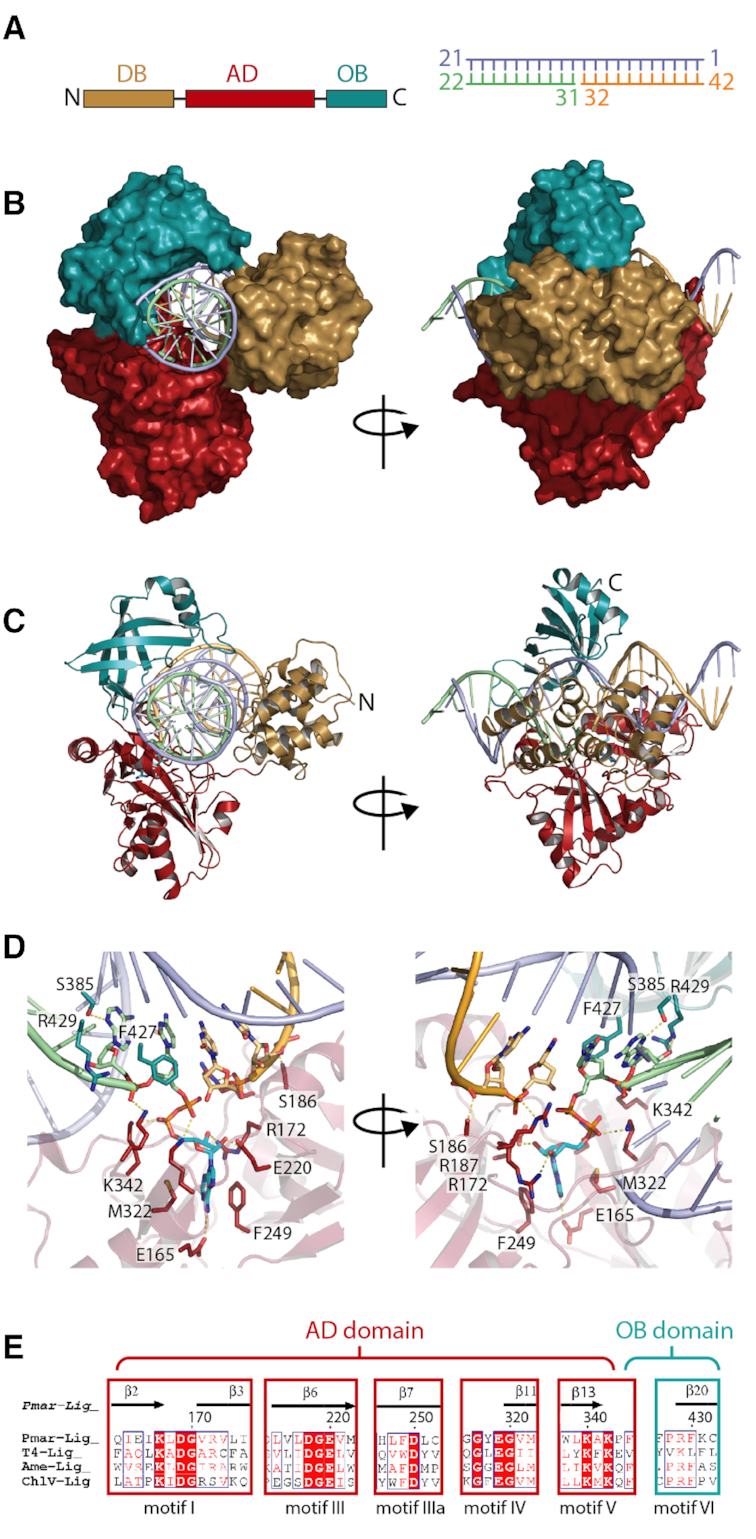
The overall structure of Pmar-Lig(PreS3). (**A**) Left: Pmar-Lig primary structure colored by domain: DNA-binding (DB, gold), adenylation (AD, red) and oligonucleotide binding (OB, green); Right: schematic of DNA substrate used for crystallization with nucleotide numbering used in the text. 3′OH strand shown in green, 5′P strand shown in orange, complement strand in purple. (**B**) Surface views of Pmar-Lig bound to substrate DNA in two orientations. (**C**) Cartoon views of Pmar-Lig bound to substrate DNA in two orientations. (**D**) Zoom-in views of interactions surrounding the nick site. (**E**) Conserved catalytic motifs of Pmar-Lig and homologs with numbering for Pmar-Lig structure.

Pmar-Lig was crystalized in complex with 21 bp DNA duplexes containing a centrally placed nick which included both the downstream 5′ phosphate and upstream 3′OH necessary for strand ligation (Figure 1A). Pre-treatment with EDTA to strip all catalytic metal ions from the enzyme produced complexes of the pre-ternary complex arrested prior to step 3 with the AMP moiety covalently linked to the 5′P of the nicked DNA (hereafter Pmar-Lig(PreS3)) to a resolution of 1.95 Å. In this configuration, the distance between the 3′O and 5′P groups of the nick is 3.5 Å with a 159° angle between the 3′O, 5′P and AMP phosphate oxygen is which is close to optimal for in-line attack.

As appears to be the archetypal configuration for many ATP-dependent DNA ligases, the three domains of Pmar-Lig form a toroidal clamp around the double-stranded DNA duplex, positioning the DNA nick in the active site, located in the central AD-domain, while the terminal DB- and OB-domains form contacts on the opposite side of the duplex, completing encirclement (Figure 1B and C).

Contacts to nucleotides in the 6-residues spanning the nick are provided by the core AD and OB domains (Figure 1D). This includes conserved interactions from the side chain of S385 to N3 of the nt33 purine base, as well as the ribose O4′ of nt34; side chain interactions from NE of R429 with both the backbone phosphate of nt34 and the O4′ of nt33 as well as stacking between F427 and the ribose ring of nt33. The side chain of R187 make ionic interactions with both the backbone phosphate oxygen and the ribose O5′ of nt31, the 3′ terminus of the nick, which receives a further hydrogen bond to its backbone from the amide nitrogen of R172. The backbone phosphates of downstream nt30 form hydrogen bonds with the side chain hydroxyl of S186 and NE2 of H234. Interactions on the complement strand opposing the nick are primarily supplied by residues from the OB domain with the exception of R120, which as described above forms polar interactions with the phosphates of nt10 and also the side chain of Q227 which hydrogen bonds to the phosphate oxygen of nt14 and the O3′ of nt15. Interacting residues in the OB domain include T415 which has side chain interactions with both the nt13 3′O and nt14 phosphate oxygens and adjacent residues S417 which contact the backbone of nt17 through side chain hydroxyl and main chain nitrogen interactions, respectively. The S383 main chain carbonyl forms a key hydrogen bond to nt12 which is base-paired to the 3′ end of the nick, and donates a second hydrogen bond from the amide nitrogen to backbone oxygens of nt13. Nucleotide 11, which is base-paired to the 5′P end of the nick receives a hydrogen bond from the side chain of T358, which also contacts the O5′ of the nt10 ribose via its main chain oxygen. Nucleotide 10 receives a further contact from ND2 of N361.

### DNA–protein interactions of Pmar-Lig

The overall structure of Pmar-Lig resembles that of other three-domain AD-ligases, and as discussed below, is strikingly similar to the recently solved T4-Lig enzyme particularly regarding the topology of its 8-helix DB-domain. As with T4-Lig, the DB-domain of Pmar-Lig represents a minimized version of the conserved helical DB-domains found in archaeal (17,18) and human ligases (8,13,19), preserving an 8-helix core, but lacking additional elements between α2/3 and α5/6 seen in these larger versions (Supplementary Figure S1).

**Figure 2.**
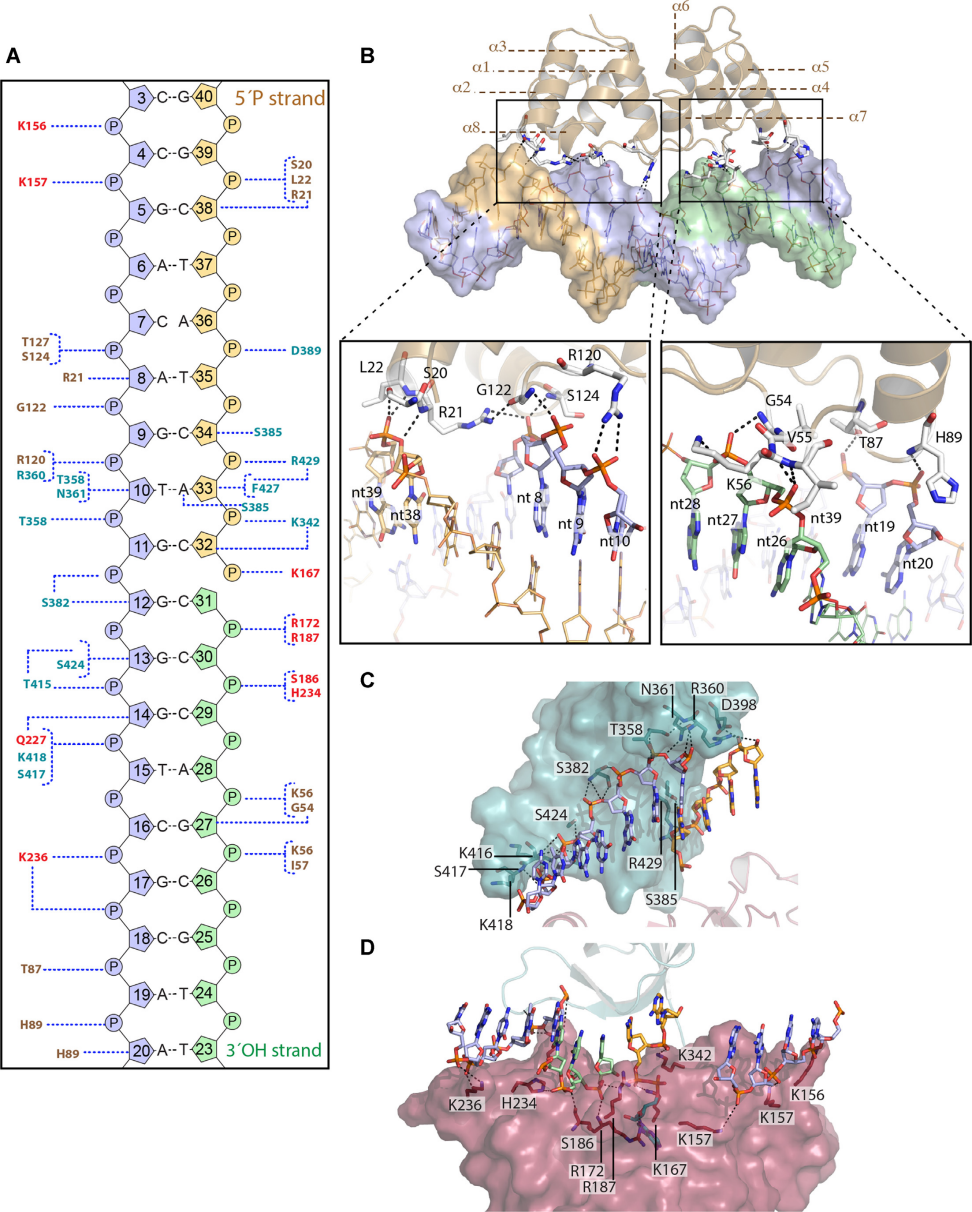
(**A**) Binding interactions of Pmar-Lig to its DNA substrate. Throughout the figure the text and structure are colored as follows: the DB-domain gold, AD-domain red and the OB-domain cyan, the 3′ OH oligo green, the 5′P oligo orange and the complement strand purple. Polar contacts are indicted by dashed lines. (A) Schematic of interactions for Pmar-Lig(PreS3) predicted by NuProPlot (30). Settings applied were: Maximum distance 4.0 Å, minimum angle 85° with contacts confirmed by manual inspection. Van der Waals interactions and water-mediated contacts are not shown. (**B**) Specific interactions between the two halves of the Pmar-Lig DBD and the substrate DNA minor groove. (**C**) electrostatic interactions between the OB domain and DNA. (**D**) Interactions between AD domain and DNA.

All three domains of Pmar-Lig make extensive contacts with the backbone of the DNA substrate producing a binding footprint of 17 nt on the complement strand and 12 nt on the broken strand; 8 nt on the 5′ side of the nick 5 nt on the 3′ side of the nick (Figure 2A). The outermost contact toward the 5′ end of the complement strand is made by the side chain of K156 of the AD domain, while the furthest downstream contacts toward the 3′ end of the complement are contributed by H89 of the DB domain which has side chain interactions with the ribose sugar of nt20 and main chain interactions with its phosphate backbone. On the nicked strand the 3′ and 5′ boundaries of the binding footprint are also defined by the DB domain. In all cases, the DNA-interacting residues of the DB domain are found in inter-helix loops with interactions on the 5′ and 3′ boundaries of the nicked strand predominately from the α1-α2 and α3-α4 loops, respectively, interactions with nt18–20 at the 3′ end of the complement from the α5–α6 loop, and nucleotides 8–10 from the α6–α7 loop (Figure 2B). The OB domain has extensive contacts with the phosphodiester backbone of the complement strand and the 5′ end of the nick (Figure 2C) while the AD domain binds both ends of the nick and more distal portions of the complement (Figure 2D). Details of the Pmar–Lig interaction with DNA are given in supplementary Table S1. These interactions with Pmar–Lig impart a classical underwound conformation to the substrate DNA as observed for other ligase–DNA structures. The extent of DNA bending and widening of the minor groove is similar to that of DNA complexes with Ame-Lig, T4-Lig and ChlV-Lig (6,7,12) and slightly less than the distortion observed with the larger mammalian ligases Hu-Lig1, Hu-Lig3 and Hu-Lig4 (8,13,19).

### Structural homology of Pmar-Lig with other AD-ligases

**Figure 3.**
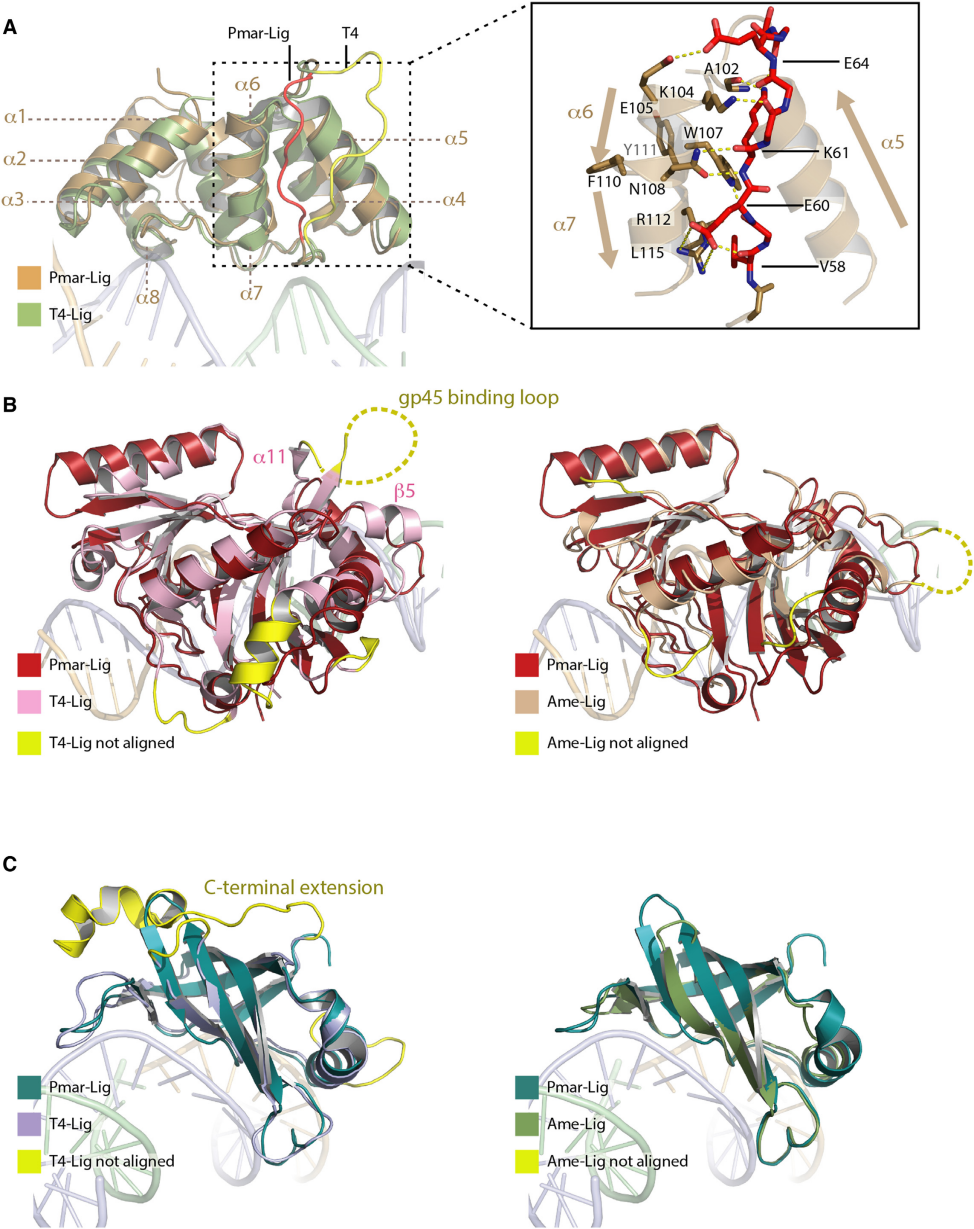
Strucutral similarity of Pmar-Lig domains with homologs T4-Lig and Ame-Lig. Yellow regions not aligned have no structural counterpart in the Pmar-Lig structure. (**A**) Comparison of DB domains of Pmar-Lig and T4-Lig. Instet shows details of the Pmar-Lig α3-α4 loop. (**B**) Comparison of AD domain of Pmar-Lig with T4-Lig (left) and Ame-Lig (right). (**C**) Comparison of OB domain of Pmar-Lig with T4-Lig (left) and Ame-Lig (right).

Superposition of the DB-domain of Pmar-Lig with that of T4 DNA ligase reveals near identical placement of helical elements with the only major difference being the position of the α3 to α4 loop which in Pmar-Lig lies between α5 and α6/7 (Figure 3A). This latter helix pair forms a continuous secondary structural element with a kink half way along its length, induced by interaction of F110 with adjacent aromatic residue Y111 as well as F39 on helix α3, W71 and F74 on helix α4 and with K8 on α2 through Van der Waals contacts. In T4-Lig these equivalent positions are substituted with smaller hydrophobic residues and without such extensive interactions, the helix is straight. The Pmar-Lig α3-α4 loop makes several main chain interactions with side chains from the kinked α6/7 helix including the V58 carbonyl to R112 NE; P59 carbonyl to W107 NE; K61 amide N to N108 ND; K61 carbonyl to N108 OD and E62 carbonyl to K104 NZ. In addition, there are several side chain interactions including a hydrogen bond between E64 and E105, a salt bridge between E60 and R112 and a hydrophobic interaction between V58 and L115. The side chain of K61 also forms a polar interaction with the main chain carbonyl of A102 in the α5-α6 inter-helix turn.

Superimposition of the core AD- and OB-domains of Pmar-Lig with T4-Lig revealed that the former lacks several secondary structural elements, including the C-terminal helical extension which decorates the OB domain of T4 and is also found in archaeal and human ligases (Figure 3C). Comparison of Pmar-Lig with Ame-Lig, also of bacterial origin albeit of a minimal type lacking a DB-domain, reveals that both have similarly spare OB domains lacking any loops or extensions compared to other ATP-dependent DNA ligases. The structures of Pmar-Lig exhibit continuous electron density along the polypeptide chain and lack disordered AD-domain loop regions equivalent those of DNA-bound ligases Ame-Lig and T4-Lig (Figure 3B). In Ame-Lig this unstructured insert located between helix α2 and β6 was not conserved in any close homologs, and was dispensable for activity demonstrated by mutagenesis (6). In T4-Lig the 26 amino acid loop between β5 and α11 binds the T4 processivity factor gp45 sliding clamp and is unnecessary for DNA ligase activity. The intrinsic absence of an equivalent interaction loop in Pmar-Lig makes it essentially a natural analogue of the T4-Lig loop-truncation mutant which was observed to crystalize more reproducibly than the wild-type (35). The lack of a gp45 interaction motif in Pmar-Lig also implies that this ligase does not bind an equivalent interaction partner in its native bacterial host.

**Figure 4.**
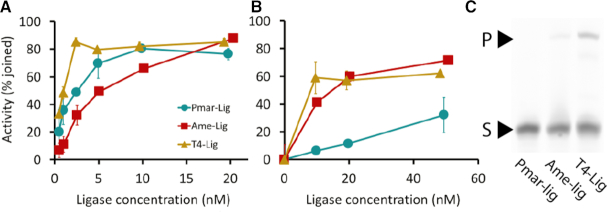
Specific activity of Pmar-Lig with different substrates compared to Ame-Lig and T4-Lig. (**A**) single-nicked substrate. (**B**) Double-strand break with 4 bp cohesive overhang. (**C**) Double-strand break without cohesive end. All activities were measured by end-point urea-PAGE assay. Percentage ligation in Panels (A) and (B) was calculated from integration of product and substrate bands. Points represent the mean of two replicate experiments; error bars represent the standard deviation from the mean. Panel (C) used an enzyme concentration of 500 nM for each enzyme.

Due to the structural similarity of Pmar-Lig with T4-Lig and Ame-Lig, it was of interest to compare the activities and substrate specificities of these three proteins. Gel-based endpoint assays reveal that although Pmar-Lig is has a similar capacity to T4 in sealing single-strand nicks and a higher specific activity than Ame-Lig, it is significantly less effective in joining double-strand break substrates the either of these homologs, being completely unable to ligate blunt ends, even after prolonged incubation times (Figure 4).

### Interdomain interactions of Pmar-Lig

**Figure 5.**
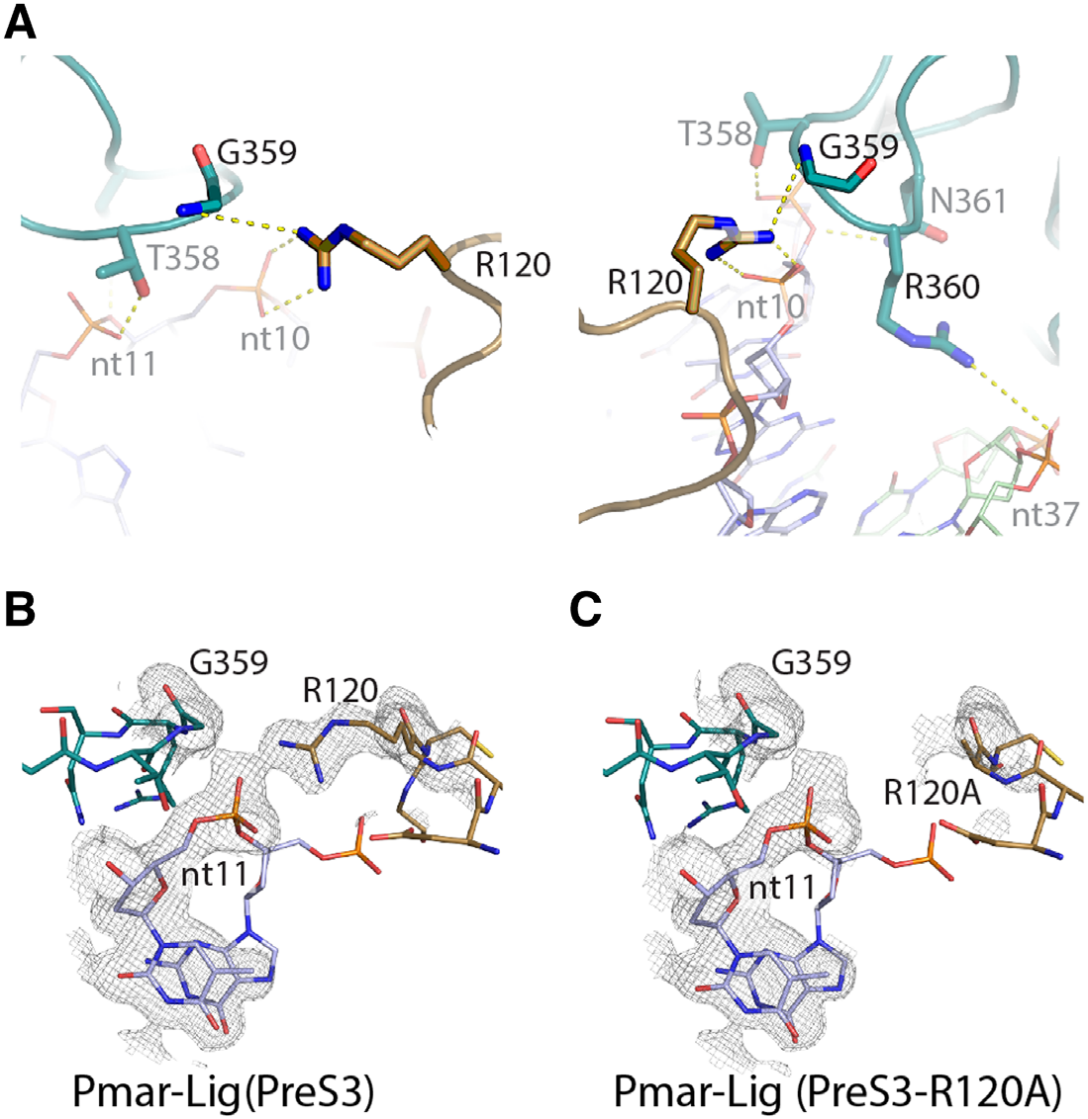
(**A**) Views of the bridging interaction between OB domain G359 and DB domain R120 and electrostatic bonds with the DNA substrate. (**B**) Observed electron density 2Fo − Fc map of wild-type Pmar-Lig(PreS3) for region around nt10, G359 and R120 carved at 1.9 Å and displayed at 1.0σ. (**C**) Observed electron density 2Fo − Fc map of R120A mutant Pmar-Lig(PreS3-R120A) for region around nt10, G359 and R120A carved at 1.9 Å and displayed at 1.0σ.

Pmar-Lig has remarkably few contacts between the three protein domains. The sole interaction between the DB- and OB-domains is a polar contact between the side chain of R120 from the α7–α8 loop and the backbone amide of G359 between β14 and β15 (Figure 5A). Both the geometry and distance are sub-optimal for hydrogen bonding indicating this interaction is weak; particularly in comparison with the salt bridge between R120 and the neighbouring backbone phosphates of the DNA. This is in contrast to T4-Lig where a lysine side chain from the OB domain forms ionic interactions with two acidic residues in the DB-domain, or the human ligases where interactions between helix α6 of the more extensive DB domain makes a network of bonds to the OB domain (Supplementary Figure S2). To investigate the importance of R120 for the activity of Pmar-Lig we generated mutants R120A and R120D. Attempts to engineer a salt bridge analogous to that in T4 were unsuccessful as the R120D/G359K double mutant was insoluble, most likely due to distortion of the OB domain loop by introduction of a large positively charged residue. The specific activity of the single mutants was unchanged relative to the wild-type (data not shown), indicating that although R120 interacts with the DNA backbone, its loss is likely compensated by the multitude of other interactions in the enzyme. Furthermore, the 2.75 Å structure of the R120A mutant in complex with the nicked DNA adenylate, also captured before S3 (hereafter PreS3-R120A), revealed that replacement of the arginine side chain had minimal impact on the overall structure relative to wild-type PreS3. This includes essentially identical conformation of the α7–α8 and β14–β15 loops, which is consistent with the specific activity results (Figure 5B and C).

Additional inter-domain interactions in Pmar-Lig include the salt-bridge between D169 of the AD-domain and R426 of the OB domain (Supplementary Figure S3A), which is conserved in almost all DNA-bound ligase structures studied to date, with the exception of T4-Lig where the arginine is replaced by phenylalanine and the hydrophobic stacking of the side chain of F143 from the C-terminal end of the DB-AD linker between F185 and F257 of the AD-domain (Supplementary Figure S3B). Two of the six cysteine residues, C145 at the N-terminal end of the AD-domain at the terminus of the interdomain linker and C332 from the β12–β13 loop are in close proximity a with Cα distance of 4.1 Å; however, the side chains are oriented away from each other and do not form a disulfide bond (Supplementary Figure S3C). Attempts to investigate any role for these residues by construction of a double C145S/C332S mutant were hindered by insolubility of the recombinant protein.

### Location of the divalent cation in a productive ligase–DNA complex

**Figure 6.**
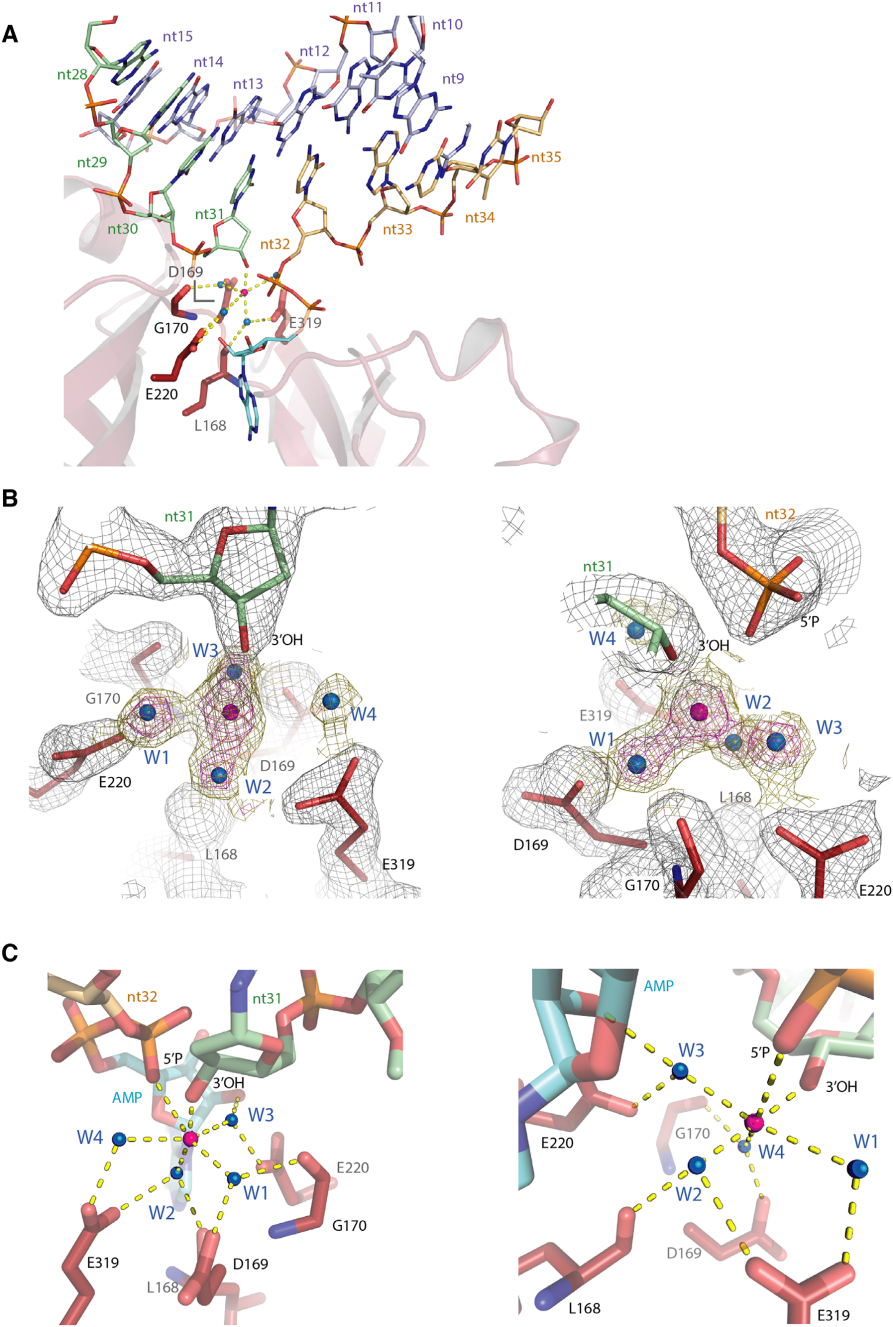
Metal binding site in Pmar-Lig(PreS3-Mn). The 5′P nucleotide is shown with light orange, the 3′OH in green, AMP with cyan and active site residues with red carbon atoms. The Mn ion is shown as a magenta sphere, coordinated water molecules as blue spheres. Electrostatic interactions are indicated by dashed yellow lines. (**A**) Pmar(PreS3-Mn) active site showing eight nucleotide streatch around the nick. Secondary structure elements of the AD domain are rendered as cartoon; other domains are omitted for clairty. (**B**) Observed electron density of the metal binding site. The 2Fo − Fc map displayed at 2.0σ carved to 1.9 Å for the nick and surrounding residues is displayed in gray mesh, and while maps carved to the Mn^2+^ and water molecules are shown at 2.0σ and 1.0σ in magenta and gold, respectively. (**C**) Two views of Mn ion interaction with the nick residues, AMP and water-mediated active site contacts highlighting the octahedral geometry of binding.

As with other polynucleotide DNA ligases, Pmar-Lig catalysis has an absolute requirement for a divalent metal cation, with Mg^2+^ producing significantly higher rates of activity than Mn^2+^ (Supplementary Figure S4A). To identify the metal binding site in a productive ligase–DNA complex that includes both 5′P and 3′OH termini, crystals of Pmar-Lig bound to 2′OMe-30 DNA substrate were soaked with MnCl_2_, which allowed modeling of a Mn^2+^ atom at the active site with an occupancy of 0.66 (Figure 6A and B). This single metal ion is coordinated with octahedral geometry by six ligands which include the 3′ terminus of the nick and one of the non-bridging phosphate oxygens of the 5′ terminus with metal-oxygen with inter atomic distances of 1.95 and 2.75 Å, respectively (Figure 6C). Water-mediated interactions provide the remaining four ligands. The first, (W1) is coordinated by the side chain of D169 and carbonyl oxygen of G170, both from motif I. The second (W2) binds the side chain of E319 (motif IV) and main chain oxygen of L168 (motif I). Water 3 (W3) contacts to the 2′OH of the AMP ribose sugar and the side chain E220 (motif III), while the final position is engaged by a delocalized water molecule with less observed electron density, water 4 (W4) that is also contacted by the side chain of E319. Interatomic distances of all contacts are provided in Supplementary Table S2.

**Figure 7.**
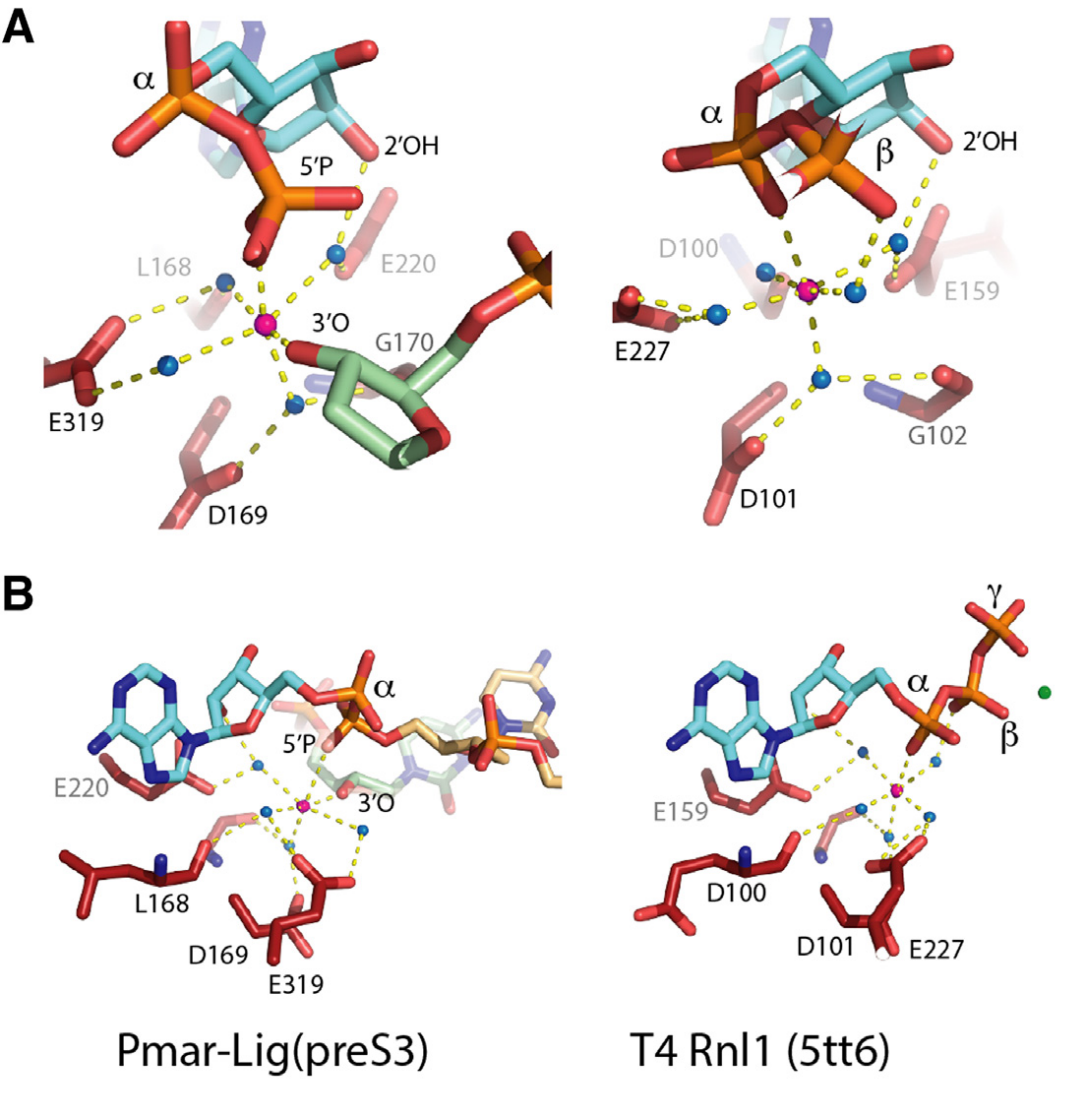
Comparison of the divalent cation coordination between the pre-step 3 structure of Pmar-Lig (left) and the pre-step 1 structure of the RNA ligase T4 Rnl1 (right; PDB ID: 5tt6). The 5′P nucleotide is shown in light orange, the 3′OH in green, AMP in cyan and active site residues in red. The catalytic metal ion is shown as a magenta sphere, coordinated water molecules as blue spheres. The non-catlaytic ion in the T4 bacteriophage RNA ligase (T4Rnal1) structure is a green sphere. Non-covalent interactions are indicated by dashed yellow lines. (**A**) View of the differing coordinating ligands contributed from the AMP cofactor and DNA substrate in the two intermediates. (**B**) View of the nucleotide orientation highlighting the comparable position of the nicked DNA strand in the Pmar-Lig structure with the β- and γ-phosphates of the AMP in T4Rnl1.

The position of this Mn ion in the Pmar-Lig(PreS3-Mn) structure directly corresponds to the site of the catalytic metal ion in the ATP-bound Michaelis complexes of RNA ligases from the T4 bacteriophage (T4Rnl1) and *Naegleria gruberi* (NgrRnl) (10,11). In the pre-step 1 complexes of T4Rnl1 and NgrRnl, the catalytic metal ion serves to deprotonate the lysine nucleophile activating it for attack on the ATP α-phosphate and stabilize the pentavalent transition state formed during step 1. Superposition of the actives sites of the RNA ligases onto Pmar-Lig(PreS3-Mn) reveals the four water-mediated contacts to the acidic side chains of motif I, III and IV as well as main chain of motif I residues and nucleoside 2′OH are structurally preserved. However, the direct interaction with the 3′ oxygen of the DNA in the Pmar-Lig(PreS3-Mn) complex replaces the water-mediated interaction with the ATP β-phosphate observed in the RNA ligase pre-Step 1 structures. Likewise, the direct Mn^2+^ contact to the DNA 5′ phosphate Pmar-Lig(PreS3-Mn) replaces the RNA ligase interaction with the AMP α-phosphate (Figure 7A). This hand over of metal interactions from the nucleoside α-phosphate pre-step 1 to the DNA 5′ phosphate pre-step 3 is consistent with an exchange of location of the chemical transformation. During step 1, a penta-coordinate transition state at the AMP α-phosphate will form during nucleophilic attack by the active site lysine, while at step 3 attack by the 3′OH will be located at the DNA 5′ phosphate; the conservation of the Pmar-Lig(PreS3-Mn) structure with the RNA ligase structures shows that in both cases the metal ion is positioned to stabilize these reactions. In contrast to step 1 lysine adenylation, which appears to be a two-metal process in ATP-utilizing nucleic acid ligases (10,11), step 3 of the ligation reaction would require only the catalytic metal ion, as the structural role of the second metal ion is functionally replaced by protein contacts to the DNA. Consistent with this, Mn^2+^ was only observed in the catalytic metal-binding site of Pmar-Lig(preS3), with the position of the ATP β- and γ-phosphates of the RNA ligase-ATP complexes being replaced by the 3′OH strand of the DNA duplex (Figure 7B).

The 1.75 Å resolution Pmar-Lig(PreS3-Mn) structure represents the first instance where the metal binding site has been unequivocally identified in a DNA-bound enzyme complex when both nick termini are intact. Previous attempts to occupy the binding site of pre-formed crystals by metal ion soaks resulted in prohibitive deterioration of crystal quality or realization of step 3 catalysis with subsequent loss of the AMP by-product (6,7). Crystallization with modified substrates containing a 3′ dideoxy nucleoside that blocks step 3 chemistry produced complexes for which the metal site could either not be populated by soaking or exhibited unusual coordination of the divalent cation which would not support catalysis (8,12,13). For example, the recently published structure of T4-Lig places a Mg^2+^ ion directly coordinated to the AMP ribose 2′OH and motif III acidic side chain with a single water-mediated contact to motif I; a position equivalent to the W3 water molecule in the Pmar-Lig(PreS3-Mn) structure (Supplementary Figure S4B). This location is too remote to effect catalysis via activation of the 3′OH, and it likely that the absence of this key ligand from the DNA precludes the metal ion occupying its native site.

### Capture of the post-step 3 intermediate

**Figure 8.**
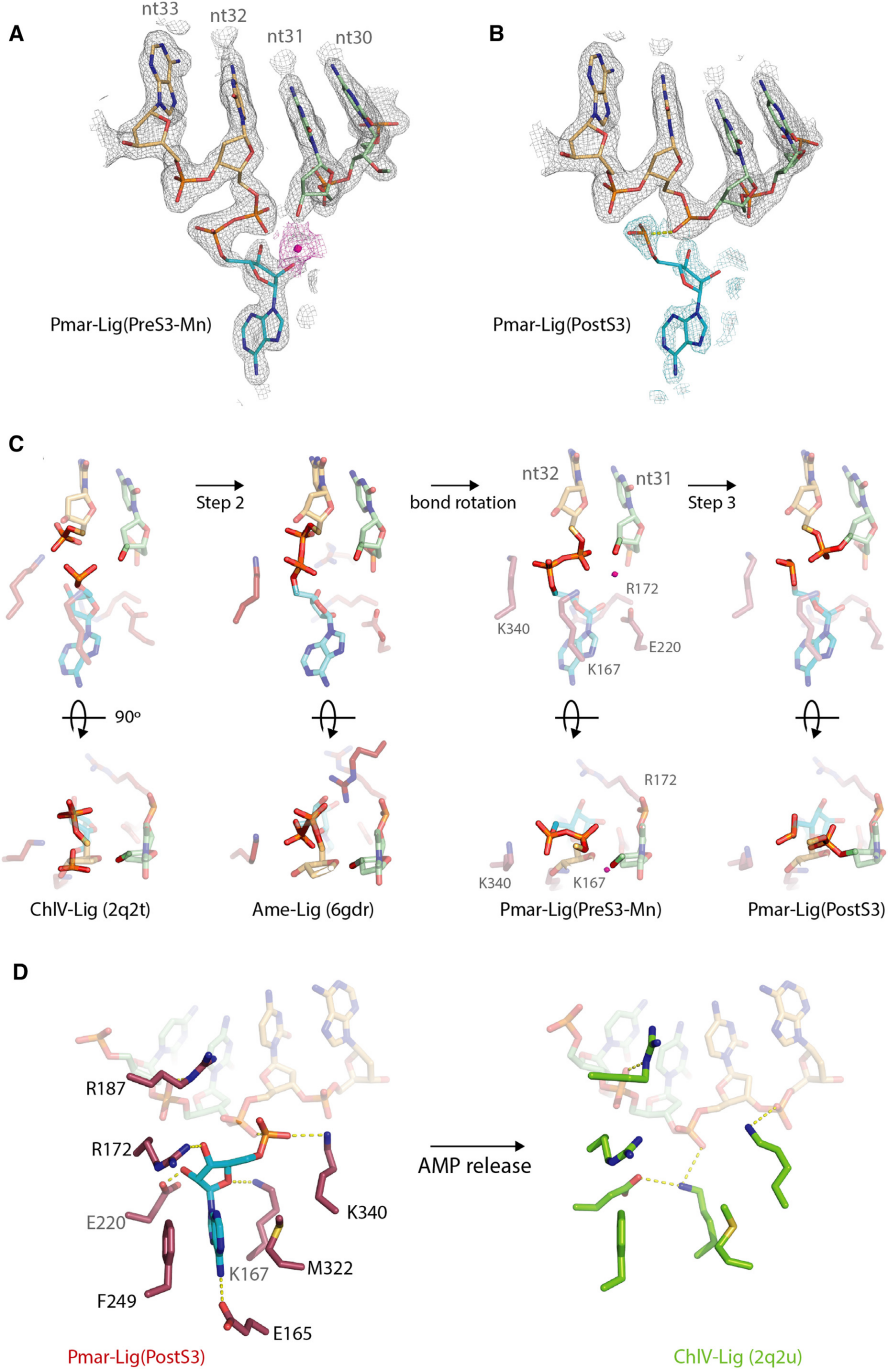
Comparison of ATP-dependent DNA ligation intermediates. The 5′P strands shown in light orange, 3′OH strand in green, AMP in cyan. Active site residues are colored as indicated on figure labels. (**A**) Observed electron density of Pmar-Lig(PreS3-Mn). The 2Fo − Fc map is shown at 2.0σ in gray mesh for the four residues of the nick and AMP cofactor. Density at 1.0σ surrounding the Mn ion in the left panel is colored magenta. (**B**) Observed electron density of the Pmar-Lig(PostS3) reaction intermediate. The 2Fo − Fc map carved to 1.9 Å around the AMP is shown at 1.0σ in cyan in the right panel overlaid with the 2Fo − Fc map at of the nucleotides and cofactor (gray). (**C**) Steps 2 and 3 intermediate in bond formation highlighting the relative orientations of the AMP α-phosphate, DNA 5′phosphate and 3′OH. In all panels the Mn^2+^ ion from the Pmar-Lig(PreS3-Mn) structure is superimposed and shown as a magenta sphere. (**D**) Conformations of the post-step 3 reactions site before and after release of the spent AMP-cofactor.

In addition to the three structures of enzyme-bound DNA-adenylate intermediate immediately prior to nick sealing (PreS3, PreS3-Mn and PreS3-R120A), a fourth complex of Pmar-Lig has been captured in the post-ternary state after step 3, with the DNA product retained in the active site, as well as residual AMP with partial occupancy. The 2Fo − Fc maps of this state, hereafter referred to as Pmar-Lig(PostS3), show continuous density between these two positions indicating the phosphodiester bond has formed between nt31 and nt32. This is in contrast to the 2Fo − Fc maps of the enzyme-adenylate forms where a clear gap is seen between the 5′P of nt32 and 3′OH of nt31 exemplified by the Pmar-Lig(PreS3-Mn) structure shown in Figure 8A.

**Figure 9.**
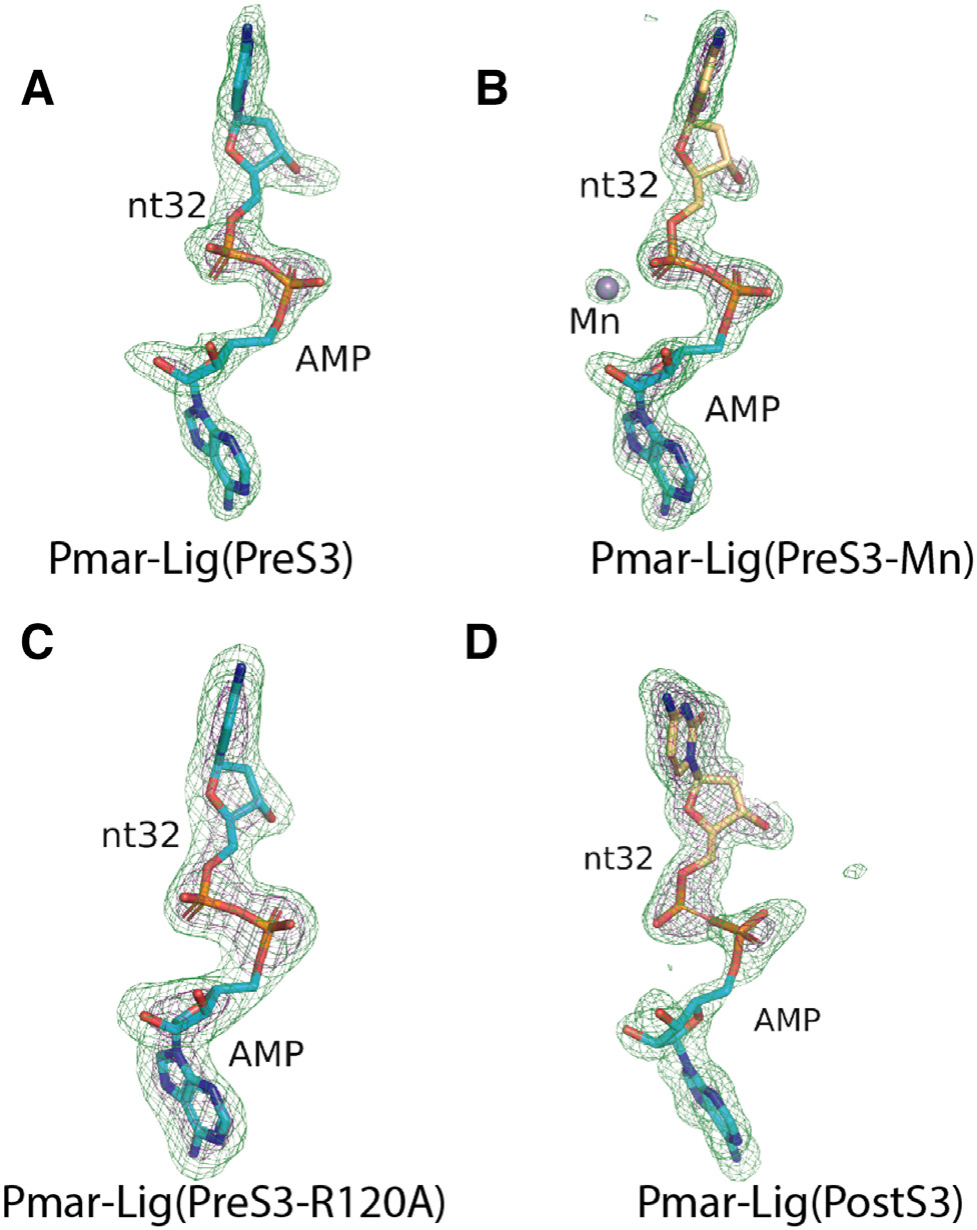
Polder OMIT maps of nucleotide 32 (nt32) and the AMP cofactor displayed at 3.5σ (green) and 6.5σ (deep purple). (**A**) Pre-step3 Pmar-Lig, (**B**) Pre-step3 Pmar-Lig with bound metal ion, (**C**) Pre-step3 Pmar-Lig R120A mutant and (**D**) Post-step3 Pmar-Lig.

The Pmar-Lig(PostS3) maps at the 1.0σ level also reveal some continuous density between the 5′ phosphate of nt32 and the α-phosphate of AMP (Figure 8B), although this is significantly less than the PreS3 structure (Figure 8A). The currently refined PDB model which most accurately represents the observed electron density places the AMP α-P-atom 1.91 Å from the O1P of nt32. This interatomic distance, which is longer than the other covalent P-O bonds (1.48–1.64 Å) of the AMP α-phosphate, coupled with the comparatively weaker electron density at this location, may be the result of population of two states; sealed DNA with only partial retention of spent cofactor, and residual unreacted adenylated DNA. In an attempt to clarify the interpretation of the AMP α-phosphate group, we calculated polder OMIT maps for all four structures (Figure 9). For the PreS3 structures electron density for the phosphoanhydride linkage is clearly visible at both the 3.5σ and 6.5σ levels and the adenine base is well defined. For Pmar-Lig PostS3 the AMP has reduced occupancy of 0.75 for the adenine nucleobase and 0.5 for the phosphate group with some continuous density to the DNA indicating covalent interaction to DNA in some portion of molecules. Attempts to accurately model an additional oxygen atom to satisfy stoichiometric requirements of the reaction after completion were unsatisfactory, likely due to the partial occupancy of this portion of the AMP moiety in the structure and possibly its mobility post step 3. Likewise, refinement with zero occupancy for the entire AMP phosphate group gave rise to significant positive difference electron density (Fo − Fc), suggesting that omitting this group entirely did not accurately represent the data either. For this reason, in final refinement, we have modeled the phosphate group without the fourth oxygen atom in to avoid overinterpretation of our data, despite acknowledging that this representation is a ‘chemical impossibility’.

In contrast to the partial occupancy of the AMP, the DNA nucleotides and most atoms of the surrounding amino acid residues have full occupancy, with the exception of the side chain of K340 of motif V. The higher occupancy of the adenine nucleoside portion of the AMP molecule (0.75) relative to the α-phosphate group (0.5) as well as the excellent density of nick nucleotides indicate that a significant proportion of the reaction has gone to completion and that some of the AMP present in the active site is the nucleotide after catalysis, thus representing an AMP-retaining post-ternary complex. Key protein-nucleotide contacts in both Pmar-Lig(PreS3) and Pmar-Lig(PostS3) include motif I K167, which has long-range ionic interactions with both an oxygen of the nick 5′ phosphate (3.4 Å) and the 4′O of the AMP ribose (3.1 Å). R172 of motif V which interacts with the AMP ribose via O3′ and E220 of motif III with contacts to ribose O2′. The AMP base is sandwiched between hydrophobic residues F249 of motif IIIa and M322 of motif IV and forms a hydrogen bond to E165 via the exocyclic N6 (Supplementary Figure S5A and B). Upon nick sealing, the distance between the 3′O and 5′P decreases from 3.4 Å to 1.6 Å when joined by the new bond. The most significant movement is in the 3′O terminus which moves 1.2 Å closer to nt32, while the 5′P terminus shifts by 0.9 Å (Supplementary Figure S5C). Notably, the sealing of the nick at step 3 does not change the DNA conformation; the two nucleotides upstream of the nick (nt12-nt30 and nt11-nt31) and one position downstream (nt10-nt32) retain the 3′endo configuration, although for the Pmar-Lig(PostS3) structure this sugar pucker is more exaggerated on the former 3′OH terminus (nt31).

### Mechanistic implications of catalytic intermediates

The elucidation of structures for both the metal-coordinated Pmar-Lig(PreS3-Mn) and AMP-occupied Pmar-Lig(PostS3) catalytic intermediates, provide considerable insight into the progress of the ligase reaction between DNA-adenylate formation and product release. The short distance between the metal ion and the 3′ terminal oxygen indicates that a key role of the divalent cation in step 3 catalysis is decreasing the pKa of the nucleophile, facilitating attack on the adjacent 5′P. In addition, the positive charge of the metal ion would serve to stabilize the pentacoordinate transition state of the 5′phosphate, formed during step 3.

An additional, structural role of the divalent cation may be to assist with reorientation of the 5′P group from its position immediately post step 2, where it occupies essentially the same location as in the enzyme-adenylate, to its catalytically relevant configuration adjacent to the 3′OH. This transition is visualized by comparison of the previously described post-step 2 intermediate of the bacterial ATP-dependent DNA ligase Ame-Lig with Pmar-Lig(PreS3-Mn) (Figure 8C), where we see the phosphodiester bond of the latter is now optimally orientated for in-line nucleophilic attack by the 3′OH.

Comparison of pre- and post-step 3 intermediates also reveals that key binding interactions between the protein and the nucleoside are essentially preserved including K167 to the nick 5′ phosphate, ribose 4′O and E220 to the ribose O2′ (Figure 4C). In contrast, the DNA-bound post-ligation intermediate of ChlV-Lig where the AMP has exited reveals a more extensive rearrangement of residues in the binding pocket. Here the ChlV-Lig K27 (Pmar-Lig K167) forms a new interaction with ChlV-Lig E67 (Pmar-Lig E220) while also retaining contacts to phosphate oxygen of nt32.

ChlV-Lig R42 (Pmar-Lig R187) remains coordinated to the phosphate oxygen of nt31 through its NE while ChlV-Lig K186 from motif V (Pmar-Lig K340) forms a new ionic interaction with the nt33 phosphate backbone. Notably, Pmar-Lig K340 which has an ionic interaction with the non-bridging oxygen from the AMP α-phosphate in Pmar-Lig(Pre-S3) has little side-chain density in the post S3 state which is consistent with greater mobility in the absence of the ridged fully occupied AMP-phosphate position. The preservation of the majority of AMP–ligase interactions subsequent to formation of the new DNA backbone phosphodiester linkage implies that it is loss of the covalent linkage to the DNA that induces expulsion of the nucleotide by-product from the binding pocket, as opposed to loss of non-covalent interactions with the protein.

The residues that are critical for water-mediated metal binding, as well as crucial AMP–protein contacts both pre- and post-step 3 are found in the well-defined motifs conserved among members of the nucelotidyl-transferase family and have been investigated by both alanine scanning and conservative mutations in previous studies (9,36–38). In the context of the present structural information the particular importance of Pmar-Lig D169 to step 3 of catalysis, inferred by its mutation in homologs from *Chlorella virus* (36) and *M. tuberculosis* (9), can be related to its role in liganding the catalytic metal ion in the pre-step 3 ternary complex (Supplementary Table S3).

## DISCUSSION

**Figure 10.**
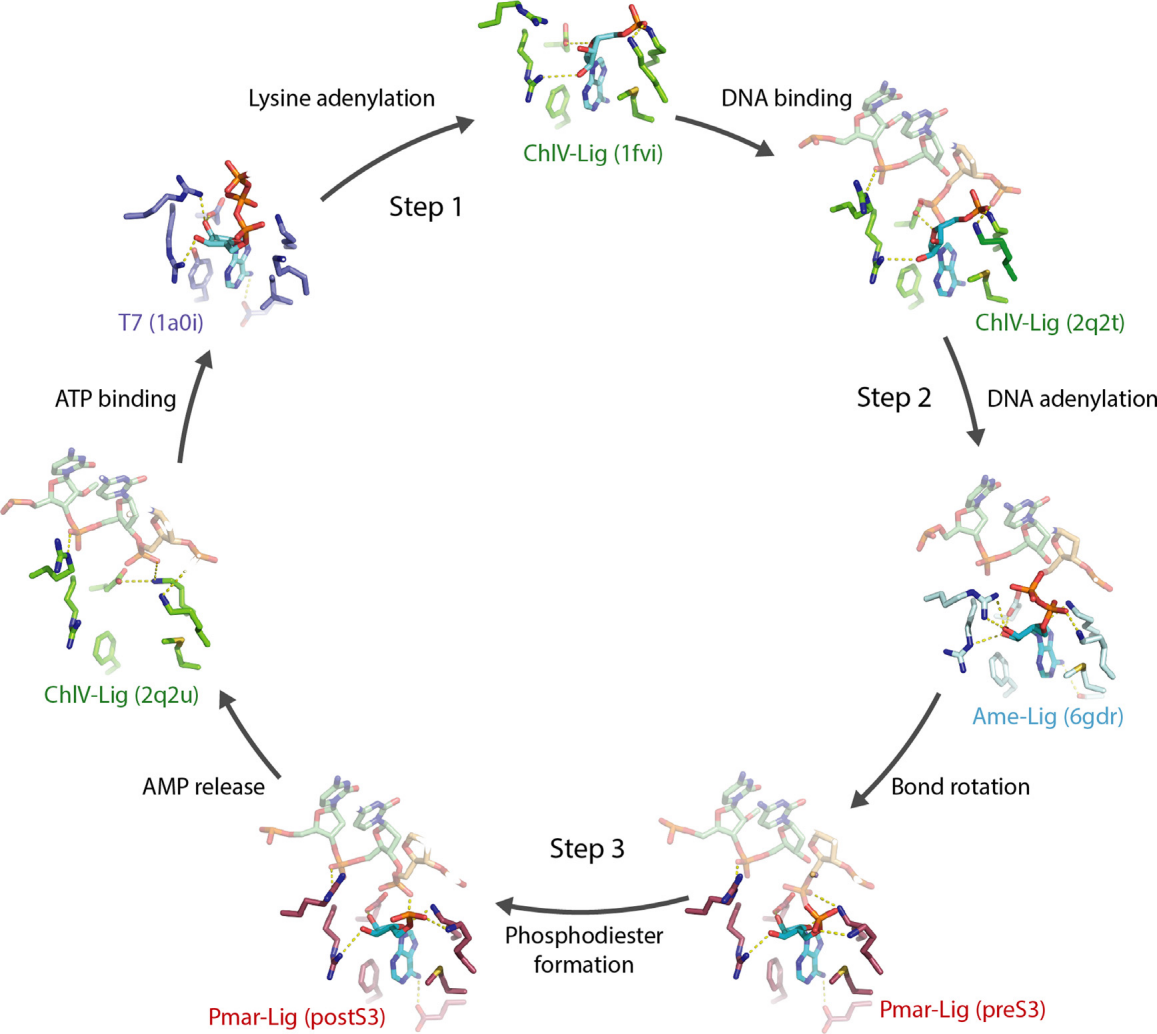
Active site conformations of ATP-dependent DNA ligases determined by X-ray crystallography at different points of the catalytic cycle. For all structures the 5′P nucleotide is shown in light orange, the 3′OH in green and the nucleotide cofactor in cyan. Active site residues are colored according to the protein as indicated in the panel lable. References in brackets are given to reaction states (present work) and individual PDB files (previously published structures).

The structural characterization of these key intermediates in the DNA ligase reaction allows reconstruction of conformational changes occurring during the catalytic cycle in near entirety (Figure 10). Previous crystallographic snapshots along the reaction trajectory included the foundational structure of the ATP-bound T7 DNA-Ligase (T7-Lig) prior to step1 enzyme-adenylate conformation which was obtained by soaking apo-protein crystals with ATP and MgCl_2_. In this state the nucleoside triphosphate is in the *syn* conformation with the α-phosphate positioned close to the side chain of the catalytic lysine (4). Remodeling of the active site post-step 1 is captured by the enzyme–adenylate structure of ChlV-Lig (5) as well as several subsequent structures (18,21,38), where a covalent linkage between the motif I catalytic lysine and the AMP α-phosphate is clear. The nucleoside now adopts the favorable *anti* conformation, a transition which is accompanied by a change in the hydrogen bonding of the motif I arginine from the ribose O2′ pre-step 1 to O3′ and loss of the interaction between a second conserved arginine (T7-Lig R55; ChlV-Lig R42) and motif III glutamate from O2′ pre-step1 to O4′ of the ribose ring. A second structure of the ChlV-Lig lysyl-Amp adduct bound to substrate DNA represents the conformation immediately preceding step 2 and reveals that polar interactions with the ribose moiety are retained, while a lysine from motif V as well as the conserved arginine contact the nicked DNA (7). The recently described ligase-bound DNA-adenylate structure of Ame-Lig defines the conformation immediately following adenylation of the 5′ phosphate of the nick, with the newly formed phosphodiester bond orientated away from the 3′OH of the nick with the 5′ phosphate of the DNA retaining its original position and the α-phosphate of the AMP forming an to interact with the motif V lysine (6). As seen in the presently described pre-step 3 structure of Pmar-Lig(PreS3-Mn) and several other DNA –adenylate complexes (8,12,13), the AMP-DNA phosphodiester bond then rotates into position for in-line nucleophilic attack by the 3′ OH group of the nick, likely assisted by the proximity of the positively charged metal ion. As the post-step 3 structure Pmar-Lig(PostS3) indicates, formation of the new inter-strand phosphodiester bond with concomitant loss of the covalent linkage to the AMP proceeds with minimal rearrangement of protein–nucleoside contacts or changes in the geometry of either this cofactor or the product DNA. Ejection of the AMP by-product is presumably spontaneous upon loss of its covalent attachment to the DNA, leaving a vacant binding pocket as delineated in a third structure of ChlV-Lig obtained by soaking crystals of the DNA-bound ligase adenylate with MnCl_2_ (7). Loss of the AMP enables the catalytic motif I lysine to form a salt bridge with the motif III glutamate, resetting the active site for receipt of a new molecule of ATP upon release of the DNA product. In the biologically relevant solution state where domain dynamics are not constrained by the crystal packing, release of AMP and DNA are believed to proceed in a concerted manner upon opening of the two catalytic domains as described in recent kinetics studies of the T4 DNA ligase (39).

Our ability to capture both the metal-liganded ligase–DNA complex and the nucleoside-bound enzyme-product complex are likely due to the fortuitous crystal packing of the Pmar–Lig DNA complex, which for the latter case would limit diffusion of the AMP out of the active site. The high degree of order in these crystals is reflected in the excellent diffraction resolutions of these complexes, which are the highest reported for DNA–ligase complexes to date and enable confident assignment of most molecular interactions in these intermediates. Formation of the DNA phosphodiester bond of the post-step 3 intermediate in the absence of exogenously added metals may be ascribed to the constrained state of the active site in the protein crystals facilitating catalysis; however, it is also possible that trace metal ions remained in the protein preparation and were sufficient to enable reaction under the lower EDTA concentration of the crystallization conditions. There were three major differences in crystallization conditions producing pre- and post-step 3 complexes: altered mother liquor composition, use of a 2′OMe-30-modified oligonucleotide for PreS3 complexes, and inclusion or omission of EDTA during incubation of Pmar-Lig with DNA. Of these factors, EDTA has a clear mechanism of inhibition, while the influence of oligonucleotide modification is less clear. A comparison of protein–DNA interactions around the methylation site shows the only structural change attributable to the presence of 2′OMe is in the side-chain of M230, suggesting its impact on activity is likely to be minimal (Supplementary Figure S6). Our use of Mn as the divalent cation for populating the metal site rather than the preferred Mg may explain why bond formation was not observed in the soaked crystals as Mn was almost 6-fold less effective in stimulating catalysis in Pmar-Lig (Supplementary Figure S4A).

It is intriguing to note that despite the considerable structural similarity between Pmar-Lig and T4-Lig, the former has a comparatively diminished capacity for joining double-strand breaks, whether cohesive or blunt. The structural basis for this difference in activity is not immediately apparent; aside from the altered position of the α3–α4 loop, the DB domain of Pmar-Lig is almost identical to that of T4-Lig and this difference does not appear to alter the DNA binding surface of the domain. Pmar-Lig lacks the 26-residue loop found in T4-Lig, which facilitates interaction with the gp45 clamp, and does not possess additional helical elements in its OB domain which are present in T4-Lig, archaeal and human DNA ligases. Although it is possible that the gp45 interaction loop contributes to double-strand break activity in T4, despite being unnecessary for nick sealing, this seems unlikely as its position is oriented away from the DNA strand. The C-terminal OB domain extension, which is absent in Pmar-Lig has a role in domain dynamics, interacting with the AD-domain in the closed DNA-free state of in *Sulfolobus solfataricus* DNA ligase, and adopting different conformations in the closed DNA-bound form of Hu-Lig3 (40,41). Again, this region is remote from the DNA binding site of the OB domain; however, differences in domain interactions may influence the respective capacities of Pmar-Lig and T4-Lig to seal double-strand breaks. A second difference in domain interactions between these otherwise highly similar ligases is the DB-OB contacts that complete encirclement of the DNA duplex. In T4-Lig this contact involves ionic interactions between acidic residues on the DB domain and a lysine residue in the OB domain, while in Pmar-Lig only a weak main chain-side chain interaction is possible; and is in fact dispensable for activity. It would be interesting to further probe these differences by mutagenesis studies and confirm the basis of double-strand break activity in T4-Lig, which underpins much of its commercial relevance as a molecular biological tool.

At last, the similarity of the core AD- and OB-domains of Pmar-Lig with the minimal Ame-Lig raises intriguing questions about the evolutionary origin of these bacterial AD-ligases. Our group has previously suggested that the minimal Lig E group of ATP-dependent DNA ligases, of which Ame-Lig is a member, arose by horizontal transfer of a viral ATP-dependent DNA ligase into a bacterium, followed by loss of the DB domain concomitant with specialization of its function in the new bacterial host (14). The present structure of Pmar-Lig, a genomic bacterial enzyme, supports this hypothesis and may represent a state similar to this postulated evolutionary intermediate.

## DATA AVAILABILITY

Atomic coordinates and structure factors for the reported crystal structures have been deposited with the Protein Data bank under accession numbers: 6RCE, 6RAR, 6RAU, 6RAS.

## Supplementary Material

gkz596_Supplemental_File
