# Supplementary material for: Structural intermediates of a DNA–ligase complex illuminate the role of the catalytic metal ion and mechanism of phosphodiester bond formation
Source: Nucleic Acids Res. 2019 Jul 17;47(14):7147–62. doi: 10.1093/nar/gkz596 (PMC6698739; doi:10.1093/nar/gkz596)
Supplement: gkz596_Supplemental_File [file gkz596_supplemental_file.pdf]

Supplementary material: Structural intermediates of a DNA-ligase complex illuminate the role of the catalytic metal ion and mechanism of phosphodiester bond formation

Adele Williamson[1, 2]*, and Hanna-Kirsti S. Leiros[1]

[1] Department of Chemistry, UiT The Arctic University of Norway, N-9037 Tromsø, Norway

[2] School of Science, University of Waikato, Hamilton 3240, New Zealand

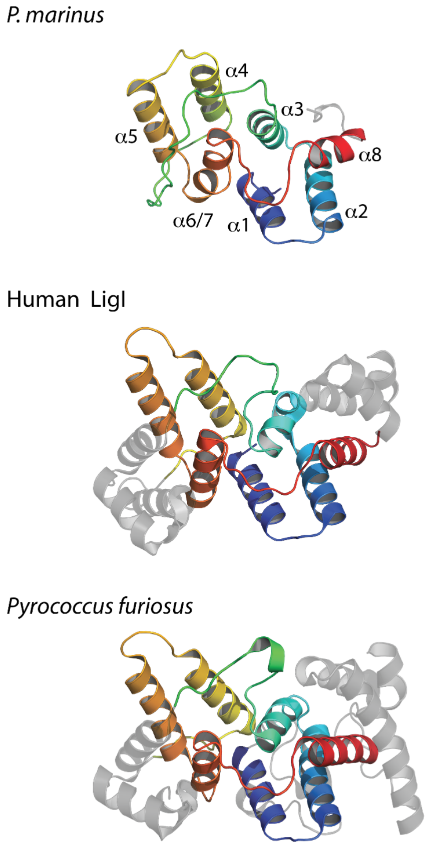

Supplementary figure S1. Comparison of DB domain of Pmar-Lig to eukaryotic and archaeal ATP-dependent DNA ligases, highlighting the conserved 7-helix domain core. Non-conserved elements in the larger ligase domains are colored grey.

Supplementary Table S1. Protein-nucleic acid interactions between Pmar-Lig(PreS3) and the DNA substrate.

Residue	Nucleotide	Atoms (Protein:DNA)	Distance (Å)
K156	nt04	Nz:OP1	3.5
K157	nt05	Nz:OP1	2.8
K157	nt05	Nz:OP2	3.6
K333	nt06	Nz:OP1	4.5
T127	nt08	OG1:OP1	2.7
R21	nt08	NH1:OP1	4.1
S124	nt08	Amide N:OP1	2.9
G122	nt09	Amide N:OP1	2.8
R120	nt10	NH1:OP2	3.3
R120	nt10	NH2:OP1	2.8
T358	nt10	Carbonyl O:O5'	3.5
R360	nt10	Carbonyl O:OP1	2.9
N361	nt10	ND2:O3'	3.1
T358	nt11	OG1:PO2	2.6
T358	nt11	Amide N:OP2	2.8
T358	nt11	OG1:OP2	2.6
S382	nt12	OG:OP1	2.9
S382	nt12	Carbonyl O:O3'	3.0
S424	nt13	OG:O3'	3.4
T415	nt13	OG1:OP1	2.8
T415	nt13	OG1:O3'	3.2
Q227	nt14	NE2:OP1	2.8
Q227	nt14	NE2:O3'	3.1
T415	nt14	OG1:OP1	2.8
K416	nt14	Amide N:OP1	3.5
Q227	nt15	NE2:OP1	2.8
S417	nt15	OG:OP2	3.5
K418	nt15	Amide N:OP2	2.9
K236	nt17	Amide N:OP1	2.9
K236	nt18	Nz:OP2	4.0
T87	nt19	OG1:OP1	3.3
H89	nt20	NE2:O3'	3.5
H89	nt20	Amide N:OP1	3.0
K56	nt27	Amide N : OP1	2.9
I57	nt27	Amide N : OP1	3.0
K56	nt28	Side chain Nz:OP1	2.8
G54	nt28	Amide N:OP2	2.8
S186	nt30	OG:OP2	2.9

H234	nt30	NE2:OP1	2.8
R187	nt31	NE:OP1	2.6
R172	nt31	Amide N:OP2	2.8
K167	nt32	Nz:OP1	3.5
K342	nt32	Nz:O3'	3.7
S385	nt33	OG:N3	3.0
R429	nt33	NE:O3'	3.0
K342	nt33	Nz:OP2	3.1
S385	nt34	OG:O4'	3.0
R429	nt34	NE:OP1	3.0
D389	nt36	Amide N:OP2	2.8
R21	nt38	Amide N : O3'	3.1
S20	nt39	OG:OP1	2.9
L22	nt39	Amide N:OP1	2.9
L22	nt39	Nz:OP2	4.3

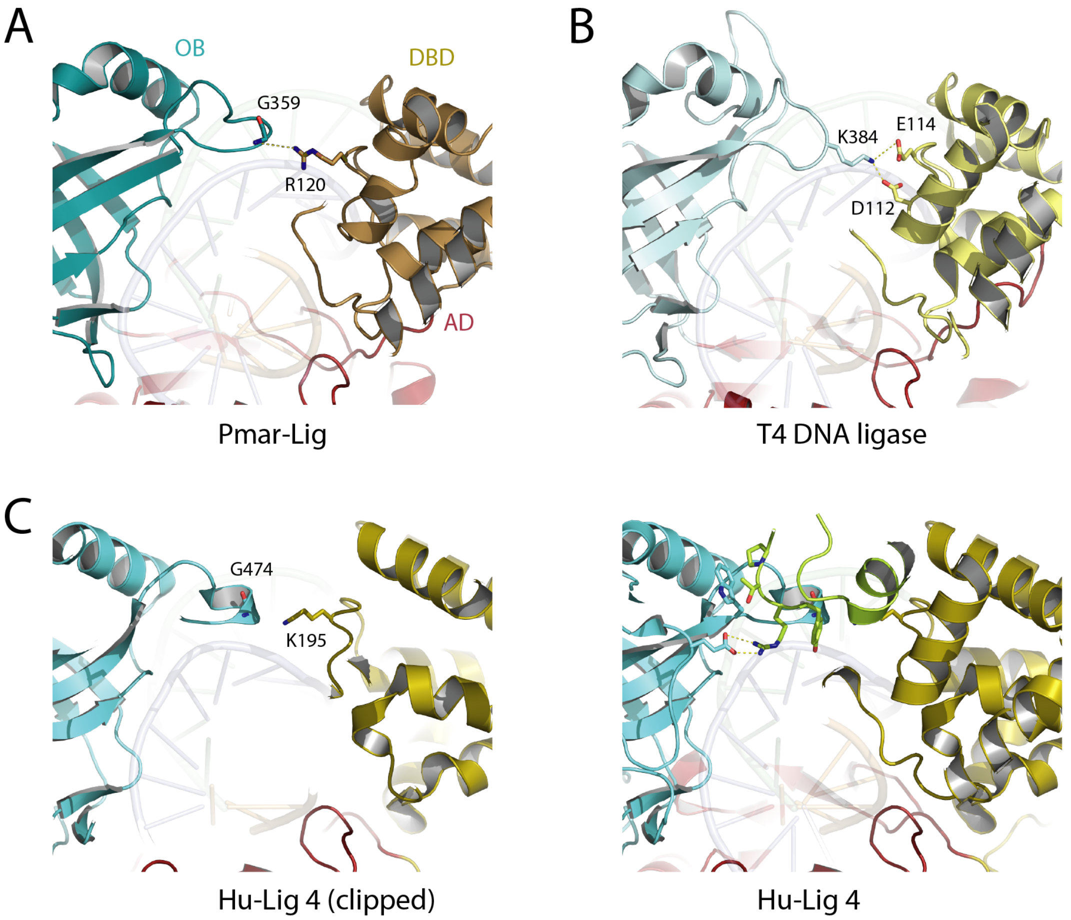

Supplementary figure S2. Comparison of DB and OB-domain interactions between A) Pmar-Lig, B) T4 and C) Hu-Lig4. The left view in panel C is clipped to show interactions between equivalent regions of the domains more clearly and enable direct comparison with architecture in A) and B). The unclipped view (right) shows the extensive interactions between the DB domain loop (lime) and the OB domain of Hu-Lig4.

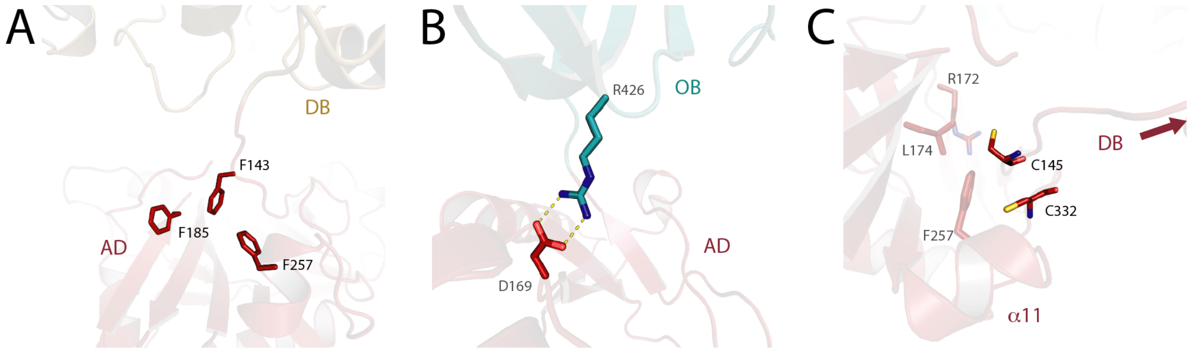

Supplementary figure S3. Inter-domain interactions of Pmar-Lig. A) hydrophobic stacking between the DB and AD domains. B) salt bridge between AD and OB domains. C) reduced cysteine pair between DB and AD domains.

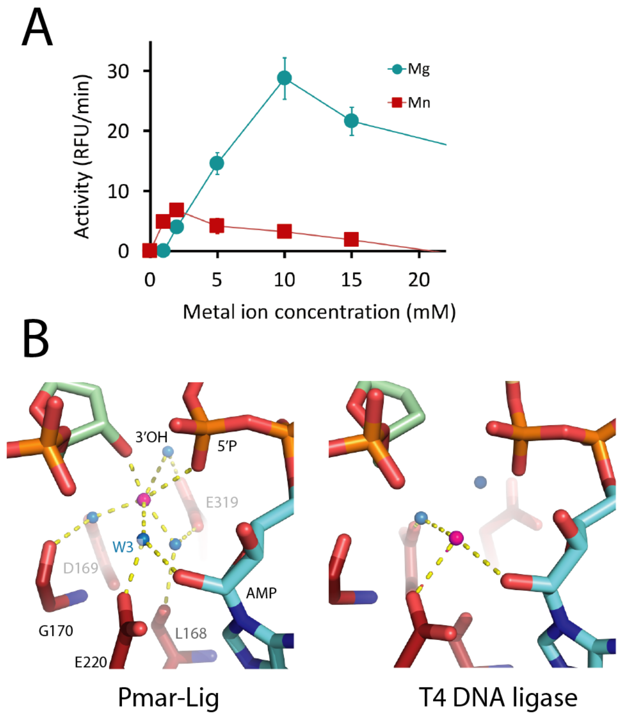

Supplementary figure S4. A) Specific activity of Pmar-Lig with different metal ions as cofactors measured by molecular beacon assay. Measurements are the mean of two replicate experiments; error bars represent the standard deviation from the mean. B) Comparison of coordinated metal ions between crystal structures of Pmar-Lig(PreS3-Mn) and T4-Lig. The 5'P nucleotide is shown in light orange, the 3'OH in green, AMP in cyan and active site residues in red. The metal ion is shown as a magenta sphere, coordinated water molecules as blue spheres. Non-covalent interactions are indicated by dashed yellow lines. Water molecule 3 (W3) in the Pmar-Lig structure which occupies an equivalent position to the Mg in T4-Lig structure is indicated.

Supplementary Table S2. Metal-ligand distances of Pmar-Lig(PreS3-Mn)

			**Distance (Å)**
**First sphere**	Mn	nt31 3'O	1.9
	Mn	nt32 5'P	2.8
	Mn	W1	2.2
	Mn	W2	2.1
	Mn	W3	2.6
	Mn	W4	2.7
**Second sphere**	W1	G170	2.7
		D169	2.2
	W2	D169	2.7
		E319	2.8
	W3	AMP 3'OH	2.7
		E220	2.2
	W4	E319	2.5

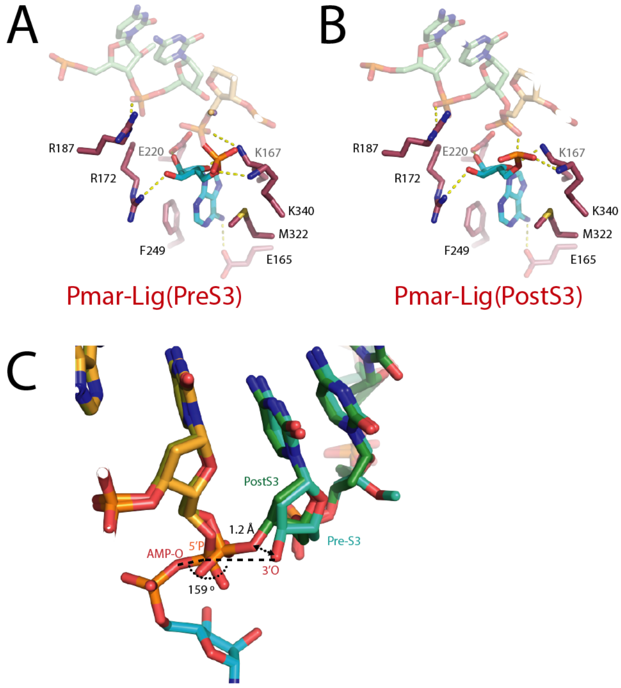

Supplementary figure S5. Catalytic site configuration of Pmar-Lig A) before and B) after step 3 catalysis. The 5'P nucleotide is shown in light orange, the 3'OH in green, AMP in cyan and active site residues in red. Non-covalent interactions are indicated by dashed yellow lines. C) superposition of nick before (cyan/ orange) and after (green/ olive) nick sealing. Distance moved by the 3'OH and angle of in-line attack are indicated.

Supplementary Table S3. Mutagenesis studies of residues from conserved motifs in homologous ATP-dependent DNA ligases.

**Motif**	**Pmar-lig**	**Position in homolg**		**Contacts in Pmar-Lig structures**	**Catalytic competence**	
		**ChlV-Lig**	**Mtu-Lig**		**Step 1**	**Step 3**
I	K167	K27	K481 [d]	AMP O4'	-	++
I	D169	D29 [a]	D483 [d]	Mn (W1)	++	-
I	R172	R32 [b]	R486	AMP O3'	-	-
Ia	R187	R42	R501 [c]	Nt31 OP	-	++
III	E220	E67 [a]	E530 [d]	Mn (W3), AMP O2'	-	-
IV	E319	E161 [a]	E613 [d]	Mn (W2, W4)	-	-
V	K340	K186	K635 [d]	AMP OP	+	++
V	K342	K188	K637 [d]		+	-

[a] Sriskanda, V. and Shuman, S. (2002) *Nucleic Acids Res*, **30**, 903-911.
[b] Sriskanda, V. and Shuman, S. (1998) *Nucleic Acids Research*, **26**, 502-531.
[c] Unciuleac, M.C., Goldgur, Y. and Shuman, S. (2019) *J Biol Chem*.
[d] Akey, D., Martins, A., Aniukwu, J., Glickman, M.S., Shuman, S. and Berger, J.M. (2006) *J Biol Chem*, **281**, 13412-13423.

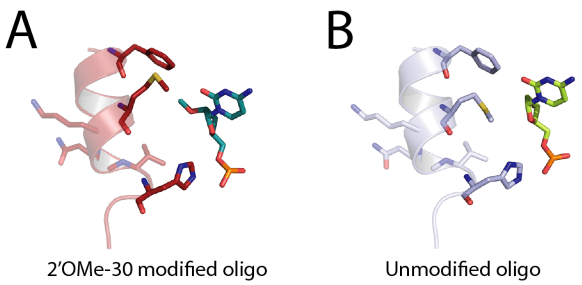

Supplementary figure S6. Side chains surrounding the C2' position on the nt30 ribose. A) Pmar-Lig(PreS3-Mn) where nt30 C2' is modified by a methyl ester. B) Pmar-Lig(PostS3) where nt30 is a standard deoxyribonucleotide.
